# Different responses of canopy and shrub leaves to canopy nitrogen and water addition in warm temperate forest

**DOI:** 10.3389/fpls.2025.1530588

**Published:** 2025-04-14

**Authors:** Mengke Li, Ruomin Sun, Yaqi He, Tenglong Zhou, Jianing Mao, Wen Li, Chang Liu, Lei Ma, Shenglei Fu

**Affiliations:** ^1^ College of Geographical Sciences, Faculty of Geographic Science and Engineering, Henan University, Zhengzhou, China; ^2^ Dabieshan National Observation and Research Field Station of Forest Ecosystem at Henan, Kaifeng, China; ^3^ Key Laboratory of Geospatial Technology for Middle and Lower Yellow River Regions (Henan University), Ministry of Education, Kaifeng, China; ^4^ Xinyang Academy of Ecological Research, Henan University, Xinyang, China

**Keywords:** canopy nitrogen addition, canopy water addition, functional trait, physiological process, metabolic process

## Abstract

**Introduction:**

Understanding the effects of nitrogen deposition and increased rainfall on plants is critical for maintaining forest ecosystem services. Although previous studies primarily examined the effects of environmental changes on leaf functional traits, the underlying physiological and metabolic processes associated with these traits remain poorly understood and warrant further investigation.

**Methods:**

To address this knowledge gap, we evaluated the influence of canopy nitrogen (25 kg ha^-1^ yr^-1^) and water (30% of the local precipitation) addition on leaf functional traits, diversity, and associated physiological and metabolic processes in the dominant species of tree and shrub layers.

**Results:**

Only the interaction between nitrogen and water significantly reduced the functional richness (FRic) of the community. The other treatments had no notable effects on functional diversity. Importantly, the physiological processes of trees and shrubs showed different regulatory strategies. In addition, there were significant changes in 29 metabolic pathways of the tree, whereas only 18 metabolic pathways were significantly altered in shrub. Among the identified metabolic pathways, four were annotated multiple times, with amino acid metabolism being the most active.

**Discussion:**

These regulatory processes enable the leaves to withstand external disturbances and maintain their relative stability under changing environmental conditions. The study findings underscore the limitations of previous research, which often relied on the direct application of treatments to the understory and so failed to accurately assess the effects of nitrogen and water on leaf functional traits. Future studies should adopt canopy-level nitrogen and water addition to better simulate the impacts of global environmental change.

## 1 Introduction

With the advancement of industrialization, the concentration of atmospheric nitrogen has significantly increased, far exceeding previously observed natural levels (Galloway et al., 2008; Ackerman et al., 2019; Stevens, 2019). From the 1980s to the early 21^st^ century, the annual average nitrogen deposition in China rose from 13.2 to 21.1 kg N per hectare per year, with expectations of continued growth (Liu et al., 2011). Under conditions of excessive nitrogen deposition, soil carbon storage can increase by 7 to 21% (Lu et al., 2021). However, increased nitrogen deposition negatively affects the growth, composition, and function of soil microorganisms (Zhang et al., 2018). Furthermore, the aboveground biomass of plants is more sensitive to nitrogen deposition than the belowground biomass (Li et al., 2015). Because of global warming, extreme precipitation events are becoming more frequent, and their intensity is increasing (O’Gorman, 2015; Myhre et al., 2019), whereas the frequency of mild-to-moderate precipitation events is decreasing (Giorgi et al., 2019). In the context of climate warming, it is crucial to consider the impact of water resource limitation on regional and global vegetation (Jiao et al., 2021). Plant biomass exhibits resistance to drought stress; however, beyond a tolerance threshold, the impact of stress becomes significant (Wan et al., 2022). Concurrent increases in nitrogen deposition and changes in precipitation occur in nature. However, research on their interactive effects is scarce.

The increase in nitrogen deposition and alterations in precipitation patterns have a significant impact on forests (Gong et al., 2004; Lu et al., 2010), with leaves being critical plant organs, demonstrating considerable plasticity in reflecting the adaptation of plants to environmental changes (Diaz et al., 2016). In the context of heightened nitrogen treatments, research in the northeastern region of China observed a reduction in leaf area quality and density, along with an increase in leaf thickness across four tree species (Khan et al., 2020). A study of seven functional groups of plants in temperate forests with increased nitrogen deposition found a significant positive correlation between leaf nitrogen content and nitrogen deposition (Roth et al., 2021). In addition, in forests limited by nutrients, nitrogen application elevates leaf nitrogen content, although changes in precipitation can constrain the productive response of leaves to added nitrogen (Lim et al., 2015). Nitrogen addition decreases the nitrogen and phosphorus absorption efficiency of *Cunninghamia lanceolata* leaves while enhancing their starch accumulation efficiency (Bu et al., 2019). Historically, most studies have concentrated on the responses of morphological traits and stoichiometric components of leaves to nitrogen deposition and precipitation changes, with minimal exploration of physiological and metabolic processes.

When plants are subjected to stress, an excessive accumulation of reactive oxygen species can cause cellular damage (Jajic et al., 2015; Żur et al., 2021). To maintain this equilibrium, plants initiate a series of responses, including the synthesis of antioxidant enzymes, osmoregulatory substances, and malondialdehyde (MDA) (Janero, 1990; del Rio et al., 1998; Jubany-Mari et al., 2009; Ren et al., 2022). Under high-temperature conditions, the levels of antioxidant enzymes and MDA in *Triticum aestivum* are significantly increased (Qi et al., 2016). *Glycine soja* can produce high levels of antioxidant enzymes under salt stress, mitigating the damage caused by reactive oxygen species (Wei et al., 2008). Research on drought and nitrogen application in the leaves of *Pinus massoniana* seedlings revealed that drought stress significantly increases the levels of MDA and proline (Pro), as well as the activity of superoxide dismutase (SOD), whereas the addition of nitrogen fertilizer can ameliorate the adverse effects of drought on the seedlings (Wang et al., 2019). These findings indicate that plant antioxidant mechanisms are self-regulated in response to environmental changes (Khaleghi et al., 2019; Guo et al., 2022). Similarly, the physiological responses of dominant forest species to nitrogen application and increased precipitation merit further exploration.

Metabolomics refers to the comprehensive study of small-molecule metabolites within an organism (Wang et al., 2020). Metabolomics data offer profound insights into the intricate relationships between metabolites and physiological conditions in response to external stimuli, such as nitrogen application or increased precipitation (Li et al., 2019b). Research on adaptational changes in *Glycine max* leaves and roots under conditions of salinity, saline-alkali stress, and drought has revealed the upregulation of genes related to calcium signaling and nucleic acid pathways under these stress conditions (Fan et al., 2013). Similarly, under nitrogen deficiency, *Glycine soja* seedlings manage to sustain relatively normal growth primarily through the regulation of key amino acids in older leaves, notably through an increase in aspartic acid and Pro metabolism (Liu et al., 2019). The assessment of metabolic processes in cold-tolerant and cold-sensitive *Hordeum distichon* at low temperatures revealed significant variations in the levels of monoacylglycerol and deoxyadenosine in the cold-tolerant variety as opposed to no notable changes in the sensitive variety (Yang et al., 2020). Metabolomics has been extensively applied to the identification and analysis of agricultural products, such as *Oryza sativa*, *Fagopyrum esculentum*, *Ipomoea batatas*, and *Raphanus sativus* (Wang et al., 2018; Li et al., 2019a; Yang et al., 2019; Zhang et al., 2020), but its application in the study of forest species remains relatively unexplored.

Despite the abundance of research on nitrogen deposition and precipitation, previous studies have predominantly focused on the morphological characteristics and stoichiometric components of plant leaves by conducting nitrogen and water addition experiments at the soil surface (Bu et al., 2019; Khan et al., 2020). This approach overlooks the internal mechanisms of plants and the interception of the canopy. Our research performed in the Jigongshan National Nature Reserve in Henan Province, China, simulated nitrogen deposition and increased rainfall to analyze changes in functional traits, physiological traits, and metabolic processes of the leaves of dominant species during the growing season. The study examined three hypotheses: 1) Nitrogen and water addition promote plant growth by altering the physiological and metabolic processes of leaves; 2) the mechanisms by which nitrogen and water influence leaves differ, and their effects interact with one another; and 3) the response of canopy tree layer leaves to nitrogen and water addition is more sensitive than that of shrub layers.

## 2 Materials and methods

### 2.1 Study site

The experimental site is located within the Jigongshan National Nature Reserve in Henan Province, spanning across coordinates 114°01’–114°06’ E and 31°46’–31°52’ N. The highest elevation in the region is 182 m. This site is situated in a transitional zone between subtropical and warm temperate climates. The region experiences an average annual temperature of 15.3°C, with an annual precipitation of 1102 mm and an average air humidity of 79%. The predominant soil type is yellow-brown earth, with a pH range of 5.0 to 6.0. Forest vegetation is distinctly divided into three layers: canopy trees, shrubs, and grasses. Human activities have led to a reduction in the original vegetation of the area, with forests now primarily consisting of plantations and secondary forests. The experimental site featured a typical mixed forest of evergreen and deciduous broadleaf trees, approximately 50 years old, with an average canopy height of 25 m and a canopy density of approximately 90% (Zhang et al., 2015).

### 2.2 Nitrogen and water addition treatments

The nitrogen deposition management involved a meticulously designed experimental platform comprising four block groups, with each group randomly allocated to four distinct treatments, with a total of 16 experimental plots. The treatments included control (CK), addition of 25 kg ha^-1^ yr^-1^ above the forest canopy (N), addition of water at 30% of the local precipitation above the forest canopy (W), and addition of nitrogen at 25 kg ha^-1^ yr^-1^ and water at 30% of the local precipitation above the forest canopy (NW). To safeguard the integrity of the experimental plots from anthropogenic disturbance, each plot was encircled by robust wire fencing. In the centers of the plots used for the N, W, and NW treatments, a 35 m tall iron tower extending 5 to 8 m above the canopy was erected. These towers were equipped with a rotating sprinkler system at their apex to ensure uniform treatment distribution across the plots (Li et al., 2021). The experimental platform was built in 2012 and began operation in April 2013.

### 2.3 Leaf sample collection

In July 2022, leaf samples were collected from six dominant species across 16 plots, including three tree species (*Liquidambar formosana* Hance.*, Quercus acutissima* Carruth., and *Quercus variabilis* Blume) and three shrub species (*Celtis sinensis* Pers.*, Lindera glauca* (Siebold & Zucc.) Blume, and *Acer buergerianum* Miq.). At least three mature individuals were selected, and 10 to 15 fully expanded healthy leaves exposed to sunlight were collected. Following collection, the leaves were meticulously cleaned with deionized water, placed in an insulated box maintained at 3 to 4°C, and promptly transported to the laboratory. A portion of the samples was preserved in a freezer at -80°C for the determination of functional traits. Another portion was treated with liquid nitrogen at -196°C for 15 min and then transported to the testing company (Novogene) using dry ice at -80°C. Three random points were selected in each sample plot, and soil samples were collected at a depth of 10 cm using a soil drill. Impurities and roots were removed from the soil, and the soil samples were sieved and brought back to the laboratory and placed in a freezer at -20°C for the determination of physical and chemical properties.

### 2.4 Laboratory sample processing

The fresh weight of leaves was determined using an electronic balance. Leaf surface area was calculated using ImageJ image processing software after scanning. The leaves were dried at 65°C for 48 h and their dry weight determined. The specific leaf area (SLA) and leaf moisture content (LMC) were calculated using Equations 1 and 2. Through two vegetation surveys in 2017 and 2022, the diameter at breast height and height of the six dominant species in the sample plots were obtained. The allometric growth equation was used to calculate the biomass. The difference showed a change in biomass over the past five years (Wen et al., 1997).


(1) $$\begin{array}{c} Specific\;leaf\;area=\frac{Leaf\;area}{Leaf\;dry\;weight} \end{array}$$

(2) $$\begin{array}{c} Leaf\;moisture\;content=\frac{Leaf\;fresh\;weight-Leaf\;dry\;weight}{Leaf\;fresh\;weight} \end{array}$$

Dried leaf samples were ground using a ball mill and weighed to obtain stoichiometric compositions. The carbon-nitrogen (C:N) ratio was determined using a vario EL cube elemental analyzer (Elementar, Langenselbold, Germany). Leaf phosphorus content (LPC) was measured by nitration with sulfuric acid, followed by the molybdenum antimony colorimetric method.

Fresh leaf samples were crushed with a mortar and weighed to generate 0.1 g samples. The extract was added to test physiological indicators (Jia et al., 2020). SOD activity in the leaves was assessed using the nitroblue tetrazolium method. Catalase (CAT) activity was determined through ultraviolet absorption. Peroxidase (POD) activity was measured using the guaiacol method. Soluble sugar (SS) content was determined by the anthrone method. Soluble protein content was determined using the Coomassie brilliant blue method. Pro content was determined by the indole trione method. MDA content was measured using the thiobarbituric acid method.

Soil moisture content (SMC) was determined using a drying method. The soil pH was determined using the potentiometric method (water-to-soil ratio of 2.5:1). The samples were extracted with KCl (2 mol/L) solution to determine the available nitrogen. Soil ammonium nitrogen (NH_4_-N) and nitrate nitrogen (NO_3_-N) were determined using the sodium salicylate method and hydrazine sulfate reduction method (Zhao et al., 2011).

### 2.5 Assessment of functional diversity

The functional richness index (FRic), functional evenness index (FEve), and functional divergence index (FDiv) were calculated using the following formulae (Equations 3–10) (Li et al., 2023b):


(3) $$\begin{array}{c} FRic=\frac{SFic}{Rc} \end{array}$$

where SFic is the ecological niche space occupied by species in the community and Rc is the ecological niche space occupied by neutral c in all communities.


(4) $$\begin{array}{c} Ew_{I}=\frac{dist\left(i,j\right)}{\left(W_{i}+W_{j}\right)} \end{array}$$

(5) $$\begin{array}{c} PEw_{I}=\frac{Ew_{I}}{\sum_{i=1}^{S-1}\min\left(Ew_{I}\right)} \end{array}$$

(6) $$\begin{array}{c} FEve=\frac{\sum_{i=1}^{S-1}\min\left(PEw_{I},\frac{1}{S-1}\right)-\frac{1}{S-1}}{1-\frac{1}{S-1}} \end{array}$$

where, S is the number of species, Wi is the relative biomass of species I, Ew is the weight uniformity, I is the branch length, EwI is the branch length weight, dist (I, j) is the Euclidean distance between species I and j.


(7) $$\begin{array}{c} \bar{dG_{I}}=\frac{\sum_{i=1}^{S}dG_{i}}{S} \end{array}$$

(8) $$\begin{array}{c} \Delta d=\sum_{i=1}^{S}W_{i}\times \left(dG_{i}-\bar{dG_{I}}\right) \end{array}$$

(9) $$\begin{array}{c} \Delta \left|d\right|=\sum_{i=1}^{S}W_{i}\times \left|dG_{i}-\bar{dG_{I}}\right| \end{array}$$

(10) $$\begin{array}{c} FDiv=\frac{\Delta d+\bar{dG_{I}}}{\Delta \left|d\right|+\bar{dG_{I}}} \end{array}$$

where *S* is the number of species, *W_i_* is the relative biomass of species *I*, $\bar{dG_{I}}$ is the average Euclidean distance from species *I* to the center of gravity, *d* is the multiplicity weight discretization, Δ*d* is the total richness weighted deviation value of species *I* from the center of gravity distance, Δ*|d|* represents the absolute richness weighted deviation.

### 2.6 Metabolomics experiments and data quality assessment

The leaf samples of 0.1 g were ground in liquid nitrogen and placed in a centrifuge tube, followed by the addition of 500 μL of 80% methanol aqueous solution. The mixture was vortex and shaken, and after standing in an ice bath, it was centrifuged at 15000 g and 4°C for 20 min. Supernatant was diluted with mass spectrometry-grade water to a methanol content of 53%, centrifuged, and the supernatant was collected for analysis using liquid chromatography-mass spectrometry (LC-MS). Equal volumes of samples were mixed from each experimental sample as quality control (QC) samples, and the QC samples were interspersed in the experimental samples for measurement to evaluate the stability of the system during the whole experimental process. The pearson correlation coefficient between QC samples was calculated based on the relative quantitative values of metabolites, and the results indicated that the quality of the sample data was satisfactory and the experiment was reliable.

### 2.7 Data analyses

SPSS Statistics 20.0 software (IBM, Armonk, NY, USA) was used to conduct one- and two-factor analyses of variance for leaf functional traits (SLA, LMC, C:N, LPC, CAT, POD, SOD, Pro, SP, SS, and MDA) and soil physical and chemical properties (SMC, pH, NH_4_-N, and NO_3_-N). The functional diversity of the leaf was calculated using R (‘FD’ package) (R Core Team, R., and Team, R.C, 2021). Amos Graphics 7.0 was used to build a structural equation model (SEM) to describe the changes in various indicators under different treatments. Graphics were produced using the Origin 2021 software.

The identified metabolites were annotated using Kyoto Encyclopedia of Genes and Genomes (KEGG), Human Metabolome (HMDB), and LIPID map databases. After the data were transformed using metaX, principal component analysis and partial least squares discriminant analysis (PLS-DA) were performed to determine the variable importance in the projection (VIP value) of each metabolite. The significance level (*P*-value) of the difference between the two groups of metabolites was calculated using the t-test, and the fold change (FC) of the difference between the two groups was calculated. Differential metabolites should meet VIP>1.0, *P*<0.05 and FC≥1.2 or FC ≤ 0.833. The figures were obtained using the Novo Cloud platform.

## 3 Results

### 3.1 Impacts of nitrogen and water addition on leaf morphological and chemical indicators

**Figure 1:**
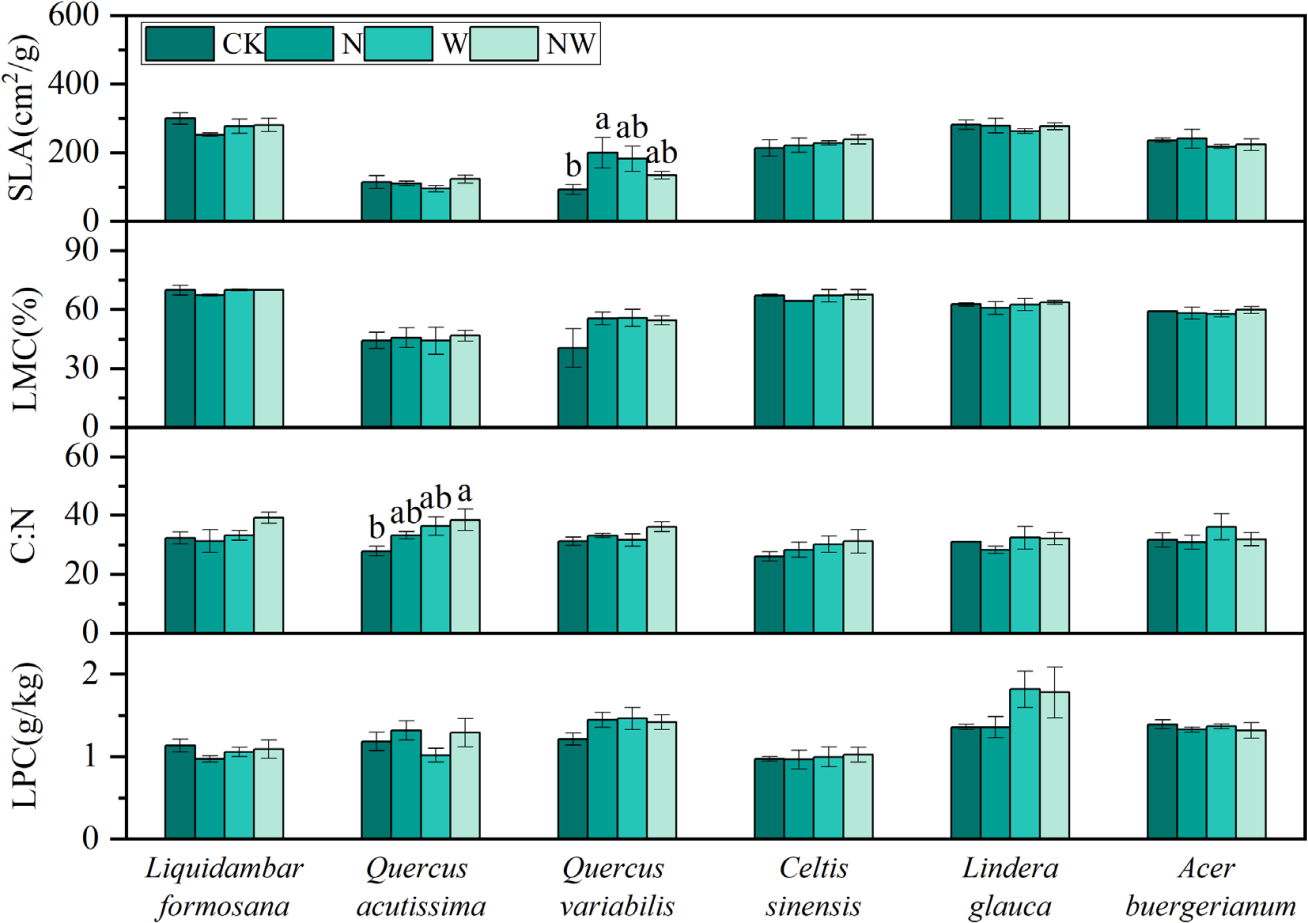
Effects of nitrogen and water addition on leaf morphological and chemical indicators. SLA, specific leaf area; LMC, leaf moisture content; C:N, the carbon-nitrogen ratio; LPC, leaf phosphorus content; CK, control; N, canopy N addition at 25 kg ha^−1^ yr^−1^; W, canopy water addition at 30% of the local precipitation; NW, canopy N addition at 25 kg ha^−1^ yr^−1^ and water addition at 30% of the local precipitation. Different lowercase letters above the error bar (standard error) indicate differences of statistical significance.

The NW treatment significantly increased the C:N in *Quercus acutissima*, while N significantly enhanced the SLA of *Quercus variabilis* (*P*<0.05). For the other species, variables such as C:N, LPC, SLA, and LMC showed no significant changes under the experimental conditions (*P*>0.05) (**Figure 1**). The results of the bifactorial variance analysis revealed a few discernible differences (**Table 1**).

**Table 1:** Effects of N, W, N * W on SLA, LMC, C:N, LPC in leaf (*P*-values).

Species	Treatment	SLA	LMC	C:N	LPC
*Liquidambar formosana*	N	0.231	0.37	0.351	0.42
	W	0.874	0.348	0.116	0.813
	N*W	0.167	0.368	0.202	0.243
*Quercus acutissima*	N	0.35	0.696	0.189	0.145
	W	0.785	0.939	0.03*	0.462
	N*W	0.23	0.908	0.543	0.601
*Quercus variabilis*	N	0.348	0.259	0.072	0.361
	W	0.712	0.247	0.301	0.282
	N*W	0.033*	0.196	0.438	0.193
*Celtis sinensis*	N	0.612	0.593	0.591	0.919
	W	0.377	0.476	0.253	0.678
	N*W	0.949	0.433	0.828	0.885
*Lindera glauca*	N	0.682	0.898	0.528	0.921
	W	0.471	0.571	0.291	0.061
	N*W	0.561	0.554	0.611	0.932
*Acer buergerianum*	N	0.766	0.793	0.434	0.357
	W	0.303	0.928	0.397	0.794
	N*W	0.976	0.498	0.578	0.908

* *P*<0.05 in the table indicate significant differences in impact; *P*>0.05 indicates no significant difference. SLA, specific leaf area; LMC, leaf moisture content; C:N, the carbon-nitrogen ratio; LPC, leaf phosphorus content; CK, control; N, canopy N addition at 25 kg ha^−1^ yr^−1^; W, canopy water addition at 30% of the local precipitation; N*W, canopy N addition at 25 kg ha^−1^ yr^−1^ and water addition at 30% of the local precipitation. Bold characters indicate statistically significant differences.

### 3.2 Impacts of nitrogen and water addition on leaf physiological processes

#### 3.2.1 Changes in physiological processes of tree leaves

**Figure 2:**
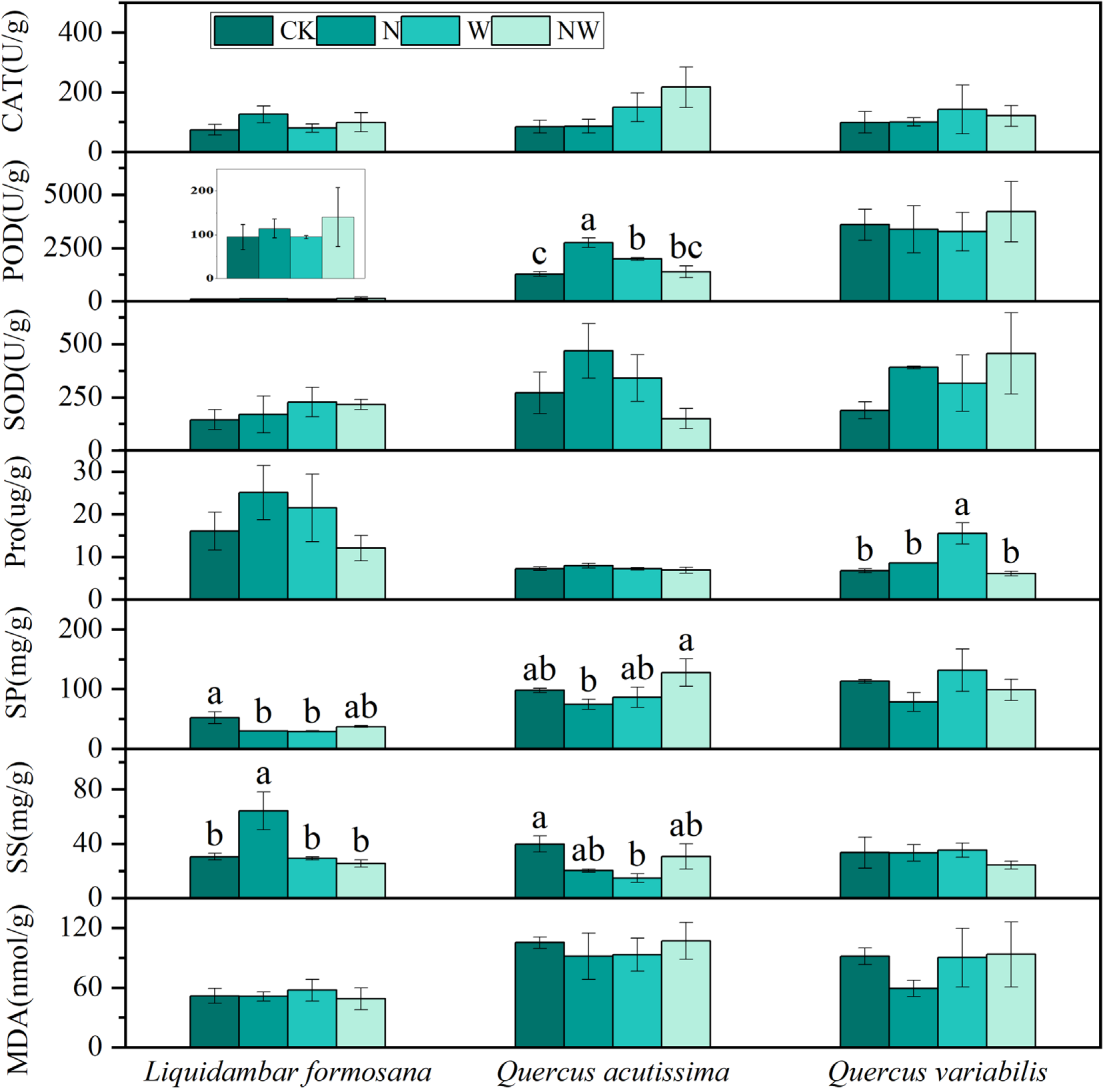
Effects of the treatments on tree leaf physiological processes. CAT, catalase; POD, peroxidase; SOD, superoxide dismutase; Pro, proline; SP, soluble protein; SS, soluble sugar; MDA, malondialdehyde; CK, control; N, canopy N addition at 25 kg ha^−1^ yr^−1^; W, canopy water addition at 30% of the local precipitation; NW, canopy N addition at 25 kg ha^−1^ yr^−1^ and water addition at 30% of the local precipitation. Different lowercase letters above the error bar (standard error) indicate differences of statistical significance.

Compared to the control group, N addition significantly enhanced POD activity in the leaves of *Quercus acutissima* and SS content in the leaves of *Liquidambar formosana* but notably reduced the SP content in the leaves of *Liquidambar formosana*. W treatment significantly increased POD activity in *Quercus acutissima* leaves and Pro content in *Quercus variabilis* leaves and significantly decreased SS content in *Quercus acutissima* leaves and SP content in *Liquidambar formosana* leaves (*P*<0.05). The NW group showed no significant changes (*P*>0.05) (**Figure 2**). The results of the two-factor analysis of variance indicated that the N treatment had a significant effect on the Pro content in *Quercus variabilis* leaves and POD activity in *Quercus acutissima* leaves. The W treatment significantly affected the Pro content in *Quercus variabilis* leaves and SS content in *Liquidambar formosana* leaves. The interaction between the N and W treatments significantly influenced the Pro content in *Quercus variabilis* leaves, SP and SS content in *Liquidambar formosana* leaves, and SS content and POD activity in *Quercus acutissima* leaves (*P*<0.05) (**Table 2**).

**Table 2:** Effects of N, W, N * W on antioxidant enzymes, osmoregulatory substances, and malondialdehyde in leaf (*P*-values).

Species	Treatment	CAT	POD	SOD	Pro	SP	SS	MDA
*Liquidambar formosana*	N	0.174	0.417	0.917	0.973	0.185	0.068	0.616
	W	0.677	0.741	0.323	0.526	0.158	0.024*	0.853
	N*W	0.518	0.741	0.766	0.147	0.017*	0.031*	0.657
*Quercus acutissima*	N	0.468	0.049*	0.974	0.686	0.569	0.771	0.998
	W	0.06	0.127	0.25	0.307	0.203	0.235	0.927
	N*W	0.481	0.001**	0.091	0.331	0.064	0.016*	0.449
*Quercus variabilis*	N	0.844	0.744	0.184	0.021*	0.155	0.454	0.54
	W	0.536	0.816	0.435	0.043*	0.391	0.622	0.487
	N*W	0.822	0.602	0.803	0.003**	0.97	0.471	0.454
*Celtis sinensis*	N	0.958	0.027*	0.709	0.148	0.495	0.136	0.25
	W	0.434	0.416	0.585	0.753	0.396	0.76	0.053
	N*W	0.542	0.782	0.594	0.599	0.52	0.274	0.051
*Lindera glauca*	N	0.965	0.915	0.626	0.004**	0.119	0.073	0.686
	W	0.004**	0.655	0.817	0.375	0.499	0.165	0.022*
	N*W	0.462	0.504	0.853	0.043*	0.141	0.594	0.91
*Acer buergerianum*	N	0.051	0.846	0.262	0.28	0.453	0.178	0.497
	W	0.001**	0.688	0.095	0.924	0.084	0.053	0.283
	N*W	0.231	0.015*	0.425	0.196	0.524	0.814	0.183

* *P*<0.05 and ** *P*<0.01 in the table indicate significant differences in impact; *P*>0.05 indicates no significant difference. CAT, catalase; POD, peroxidase; SOD, superoxide dismutase; Pro, proline; SP, soluble protein; SS, soluble sugar; MDA, malondialdehyde; CK, control; N, canopy N addition at 25 kg ha^−1^ yr^−1^; W, canopy water addition at 30% of the local precipitation; N*W, canopy N addition at 25 kg ha^−1^ yr^−1^ and water addition at 30% of the local precipitation. Bold characters indicate statistically significant differences.

#### 3.2.2 Changes in physiological processes of shrub leaves

**Figure 3:**
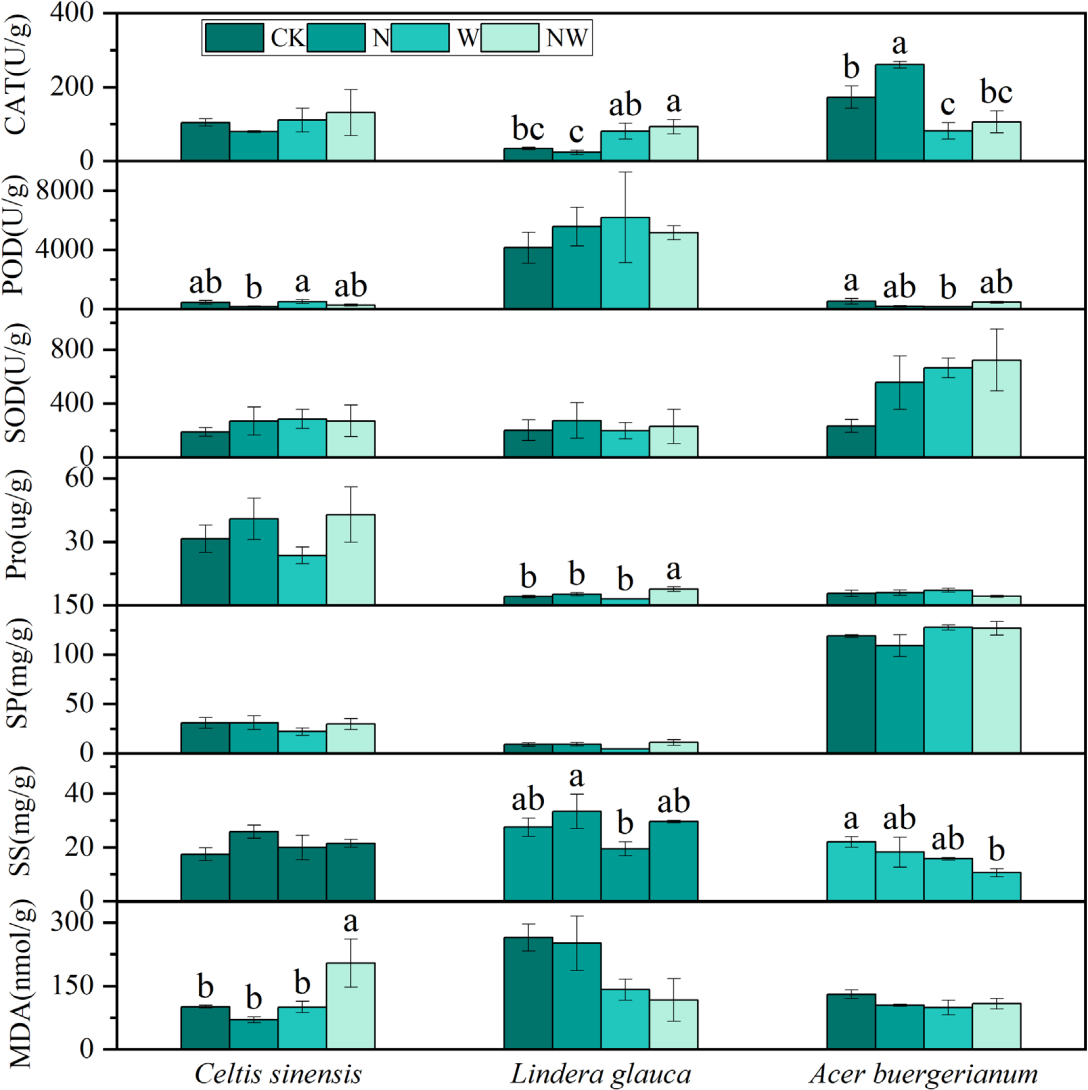
Effects of the treatments on shrub leaf physiological processes. CAT, catalase; POD, peroxidase; SOD, superoxide dismutase; Pro, proline; SP, soluble protein; SS, soluble sugar; MDA, malondialdehyde; CK, control; N, canopy N addition at 25 kg ha^−1^ yr^−1^; W, canopy water addition at 30% of the local precipitation; NW, canopy N addition at 25 kg ha^−1^ yr^−1^ and water addition at 30% of the local precipitation. Different lowercase letters above the error bar (standard error) indicate differences of statistical significance.

The N treatment significantly enhanced CAT activity in *Acer buergerianum*, while the W treatment significantly reduced CAT and POD activities in *Acer buergerianum*. NW treatment significantly increased CAT activity and Pro content in *Lindera glauca*, as well as MDA content in *Celtis sinensis*, but decreased the SS content in *Acer buergerianum* (**Figure 3**). A bivariate analysis of variance revealed the significant effect of the N treatment on Pro content in *Lindera glauca* and POD activity in *Celtis sinensis*. The W treatment significantly affected the MDA content and CAT activity in *Lindera glauca* and CAT activity in *Acer buergerianum*. The combined NW treatment had a significant effect on Pro content in *Lindera glauca* and POD activity in *Acer buergerianum* (**Table 2**).

### 3.3 Influence of nitrogen and water addition on forest parameters

#### 3.3.1 Response patterns of community functional diversity

**Figure 4:**
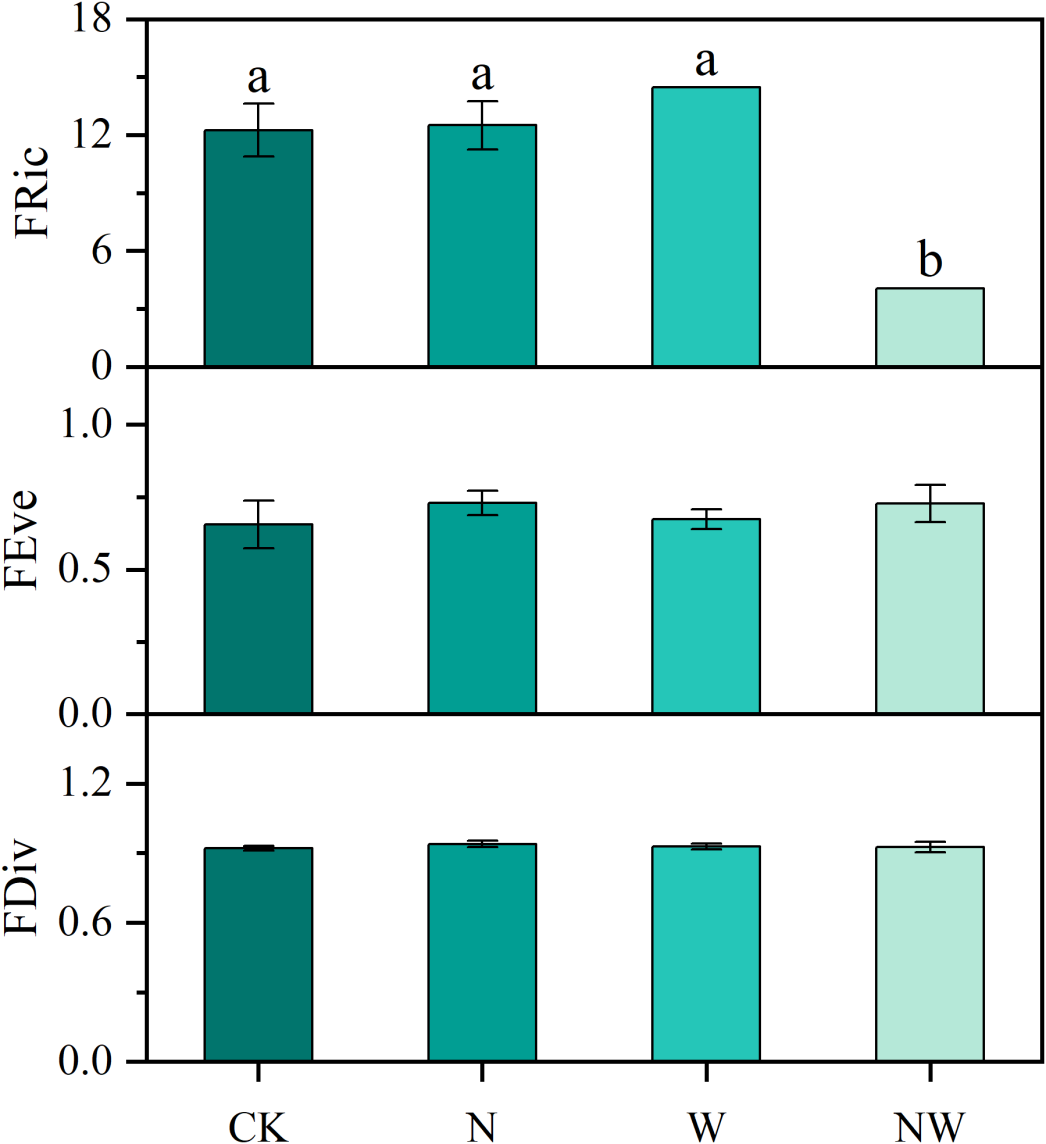
Effects of nitrogen and water addition on functional diversity. N, canopy N addition at 25 kg ha^−1^ yr^−1^; W, canopy water addition at 30% of the local precipitation; NW, canopy N addition at 25 kg ha^−1^ yr^−1^ and water addition at 30% of the local precipitation. Different lowercase letters above the error bar (standard error) indicate differences of statistical significance.

Compared with the CK treatment, in the NW treatment there was a significant decrease in leaf FRic (*P*<0.05), with no other notable changes observed in the N and W treatments. In addition, both FEve and FDiv remained stable under nitrogen and water addition (**Figure 4**).

#### 3.3.2 Response pathways of tree and shrub samples

**Figure 5:**
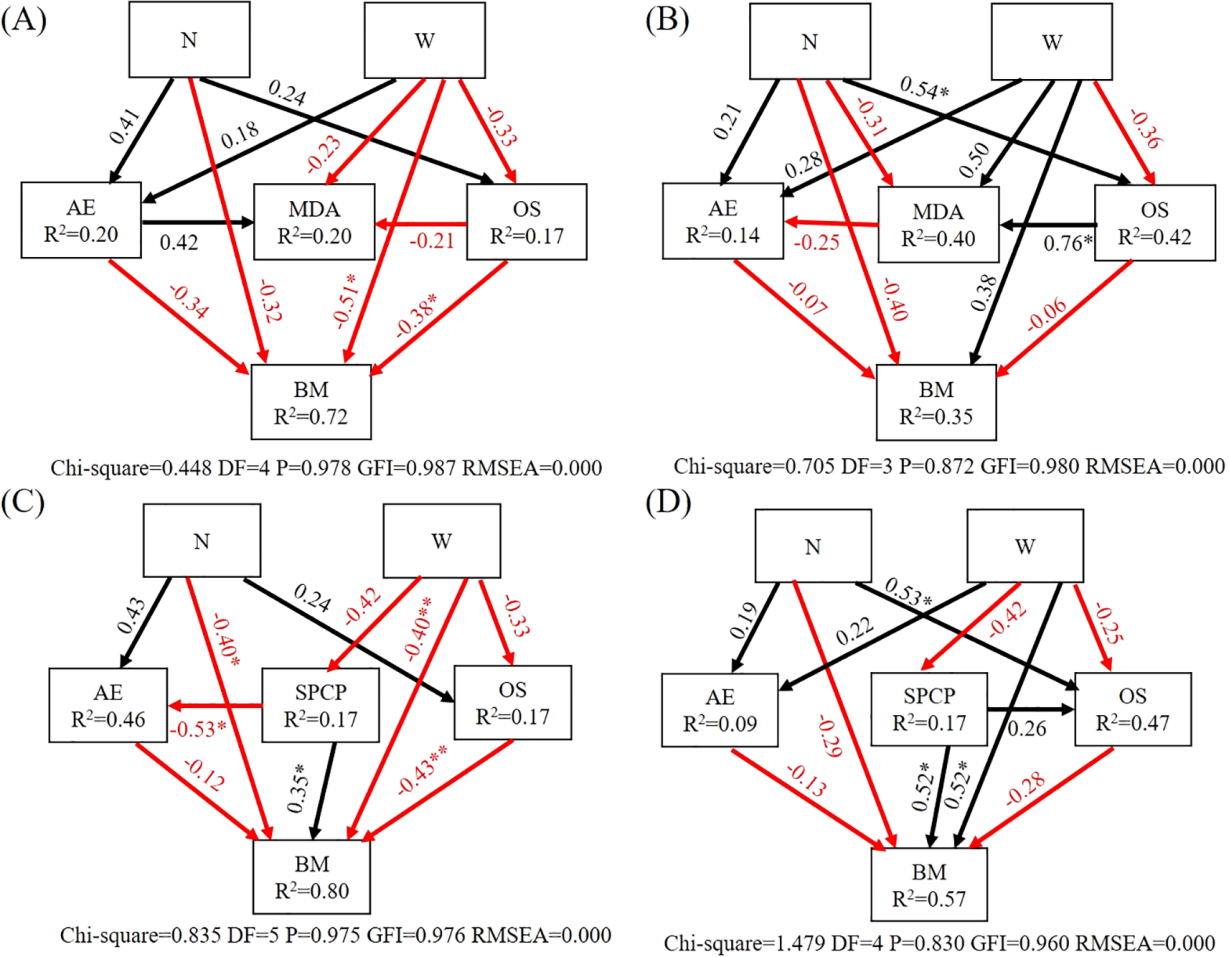
Structural equation model analysis of the influence pathways of nitrogen and water addition. **(A)** Structure equation model of tree. **(B)** Structure equation model of shrub. **(C)** Structure equation model of tree and soil. **(D)** Structure equation model of shrub and soil. N, canopy N addition at 25 kg ha^−1^ yr^−1^; W, canopy water addition at 30% of the local precipitation; AE, antioxidant enzyme; OS, osmoregulation substance; MDA, malondialdehyde; BM, biomass; SPCP, soil physical and chemical properties; *, *P<* 0.05. **, *P<* 0.01. The model fitting data are shown in the lower of the pathway figure. The black arrows represent positive effects, and the red arrows represent negative effects. Values associated with the arrows represent standardized path coefficients. R^2^ values associated with the response variables indicate the proportion of variation explained by relationships with other variables.

Within the tree SEM (**Figure 5A**), the W treatment had a significant negative impact on tree biomass. The content of osmoregulatory substances had a significant negative effect on tree biomass. In shrub SEM (**Figure 5B**), the N treatment significantly positively influenced the osmoregulatory substances in shrub leaves, and osmoregulatory substances had a significant positive effect on MDA (*P*<0.05).

#### 3.3.3 Response pathways of tree, shrub, and soil samples

The N, W, and NW treatments significantly increased the soil pH. W and the interaction between N and W had a significant effect on the pH (*P*<0.05). In addition, there were no significant changes in soil moisture, NH_4_-N, and NO_3_-N contents (**Supplementary Figure S1**, **Supplementary Table S1**).

In the tree and soil SEM (**Figure 5C**), the N and W treatments had a significant negative effect on tree biomass. Soil physical and chemical properties had a significant negative effect on the antioxidant enzymes of tree leaves and a significant positive effect on biomass. Osmoregulatory substances had a significant negative effect on biomass. In the shrub and soil SEM (**Figure 5D**), the N treatment had a significant positive effect on shrub leaf osmoregulatory substances, the W treatment had a significant positive effect on shrub biomass, and soil physical and chemical properties had a significant positive effect on biomass (*P*<0.05).

### 3.4 Impacts of nitrogen and water addition on leaf metabolic processes

#### 3.4.1 Overview of metabolic products

Using HMDB data to annotate metabolites in leaf samples, we identified 980 and 1353 metabolites in trees and shrubs, respectively. The metabolic types of trees and shrubs were similar. These predominantly include lipids and lipid-like molecules, phenylpropanoids and polyketides, organoheterocyclic compounds, organic acids and derivatives, benzenoids, along with organic oxygen compounds. This array of metabolites underscores the fundamental metabolic processes inherent to plants (**Supplementary Tables S2**, **S3**).

#### 3.4.2 Filtering and analysis of differential metabolites

**Figure 6:**
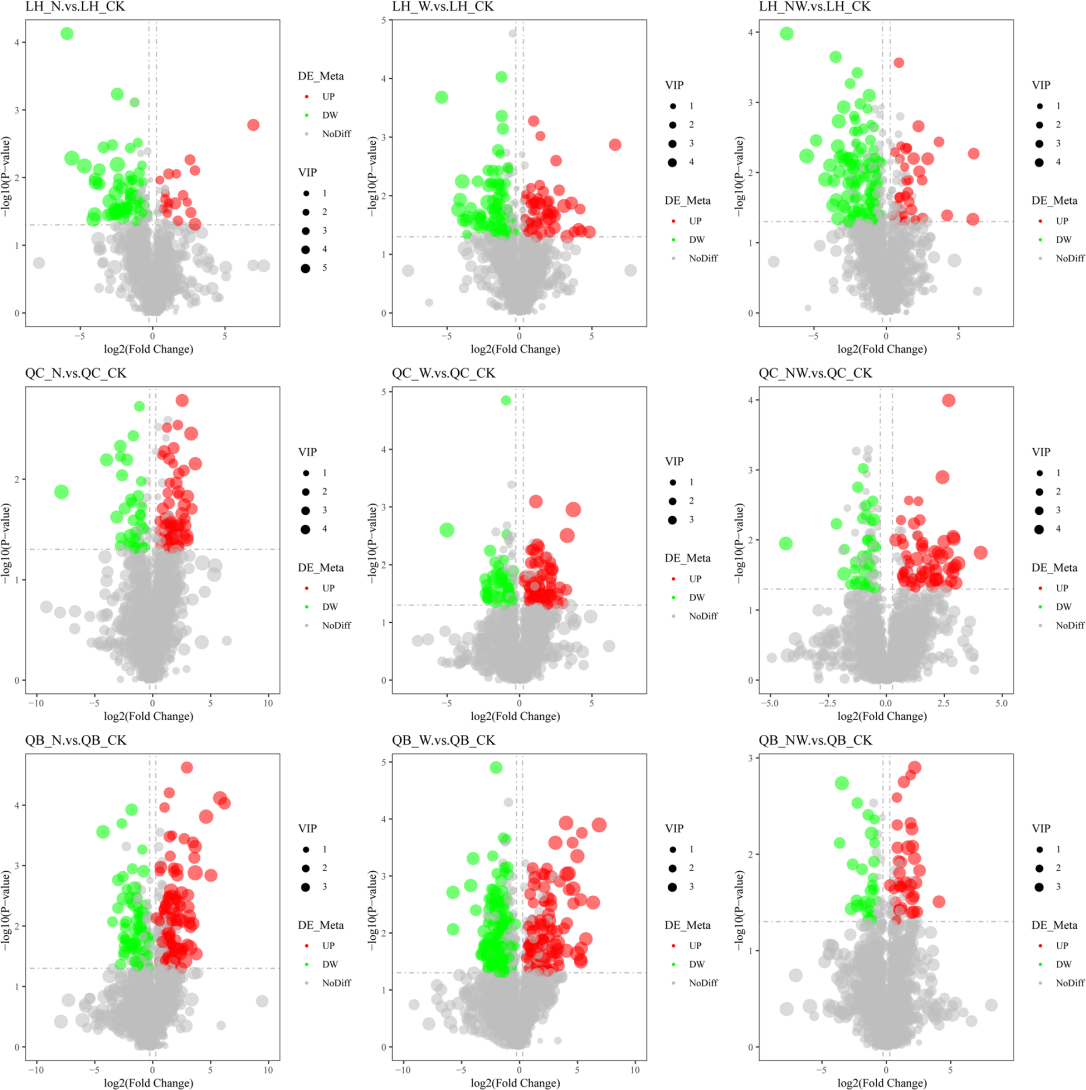
Volcano map of differential metabolites of tree leaf under nitrogen and water addition. LH, *Liquidambar formosana* Hance.; QC, *Quercus acutissima* Carruth.; QB, *Quercus variabilis* Blume.; CK, control; N, canopy N addition at 25 kg ha^−1^ yr^−1^; W, canopy water addition at 30% of the local precipitation; NW, canopy N addition at 25 kg ha^−1^ yr^−1^ and water addition at 30% of the local precipitation. Significantly up-regulated metabolites are represented by red dots, significantly down-regulated metabolites are represented by green dots, and the size of the dot represents the VIP value.

**Figure 7:**
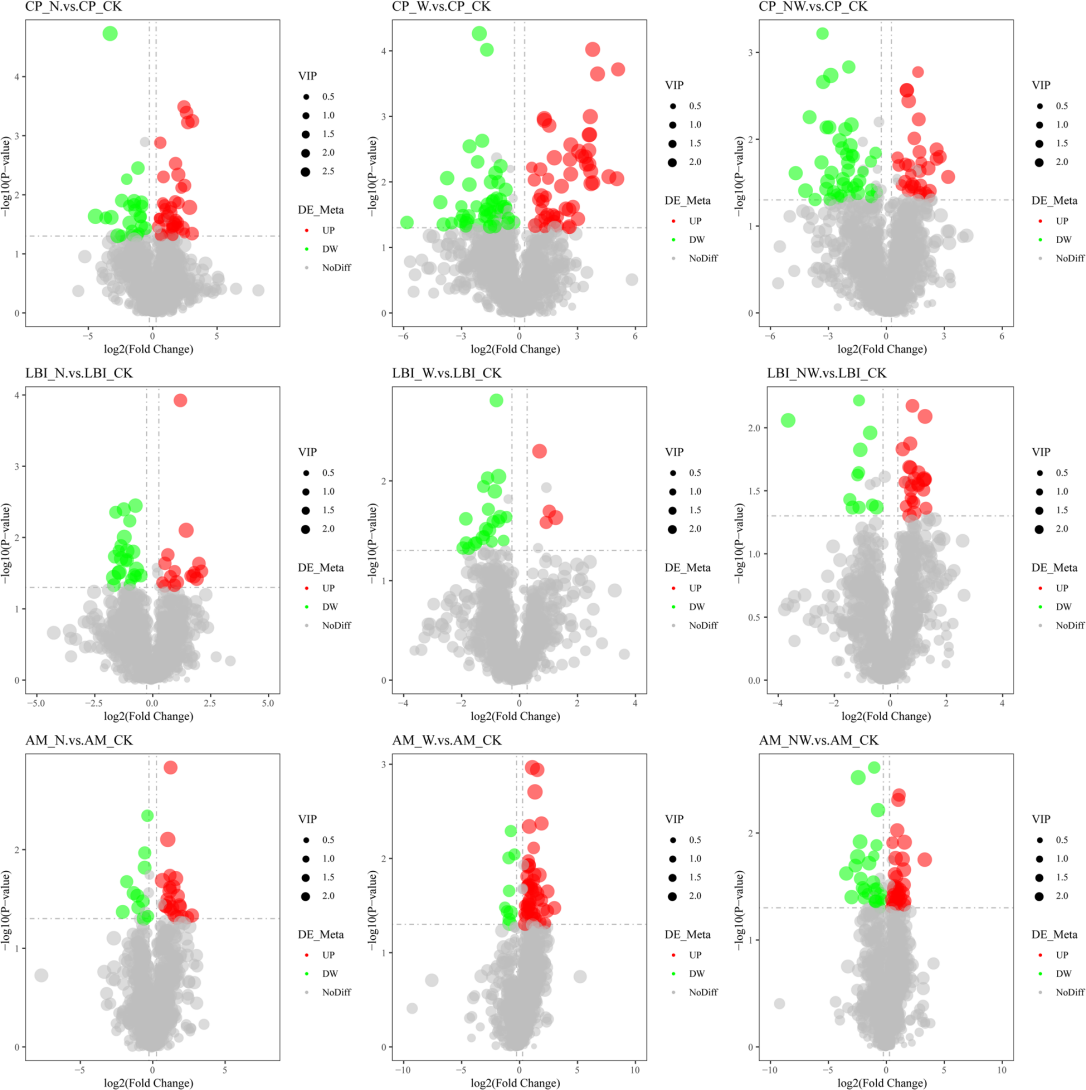
Volcano map of differential metabolites of shrub leaf under nitrogen and water addition. CP, *Celtis sinensis* Pers. LBI, *Lindera glauca* (Siebold & Zucc.) Blume; AM, *Acer buergerianum* Miq.; CK, control; N, canopy N addition at 25 kg ha^−1^ yr^−1^; W, canopy water addition at 30% of the local precipitation; NW, canopy N addition at 25 kg ha^−1^ yr^−1^ and water addition at 30% of the local precipitation. Significantly up-regulated metabolites are represented by red dots, significantly down-regulated metabolites are represented by green dots, and the size of the dot represents the VIP value.

Among the leaves of the six species, compared to the control group, there were 484 metabolites identified in the N treatment, with 262 metabolites being upregulated and 222 being downregulated. In the W treatment, 666 differential metabolites were observed, comprising 320 upregulated and 346 downregulated metabolites. In the NW treatment, 474 differential metabolites were identified, of which 221 were upregulated and 253 were downregulated (**Table 3**; **Figures 6**, **7**). In the N treatment, most leaf metabolites of *Quercus acutissima*, *Quercus variabilis*, *Celtis sinensis*, *Acer buergerianum* were upregulated, while most leaf metabolites of *Liquidambar formosana* and *Lindera glauca wer*e downregulated. In the W treatment, most leaf metabolites of *Quercus acutissima* and *Acer buergerianum* were upregulated, while those of *Liquidambar formosana*, *Quercus variabilis*, *Celtis sinensis*, *Lindera glauca* were downregulated. In the NW treatment, most leaf metabolites of *Quercus acutissima*, *Quercus variabilis*, *Lindera glauca*, *Acer buergerianum* were upregulated, whereas those of *Liquidambar formosana* and *Celtis sinensis* were mostly downregulated.

**Table 3:** Effects of the treatments on leaf differential metabolites.

Species	up/down	N.vs.CK	W.vs.CK	NW.vs.CK
*Liquidambar formosana*	up	18	51	35
	down	64	80	112
*Quercus acutissima*	up	59	61	58
	down	33	41	33
*Quercus variabilis*	up	108	104	37
	down	67	142	27
*Celtis sinensis*	up	39	51	31
	down	24	52	43
*Lindera glauca*	up	16	4	24
	down	23	22	11
*Acer buergerianum*	up	22	49	36
	down	11	9	27

CK, control; N, canopy N addition at 25 kg ha^−1^ yr^−1^; W, canopy water addition at 30% of the local precipitation; N*W, canopy N addition at 25 kg ha^−1^ yr^−1^ and water addition at 30% of the local precipitation.

#### 3.4.3 KEGG annotation and enrichment analysis

**Figure 8:**
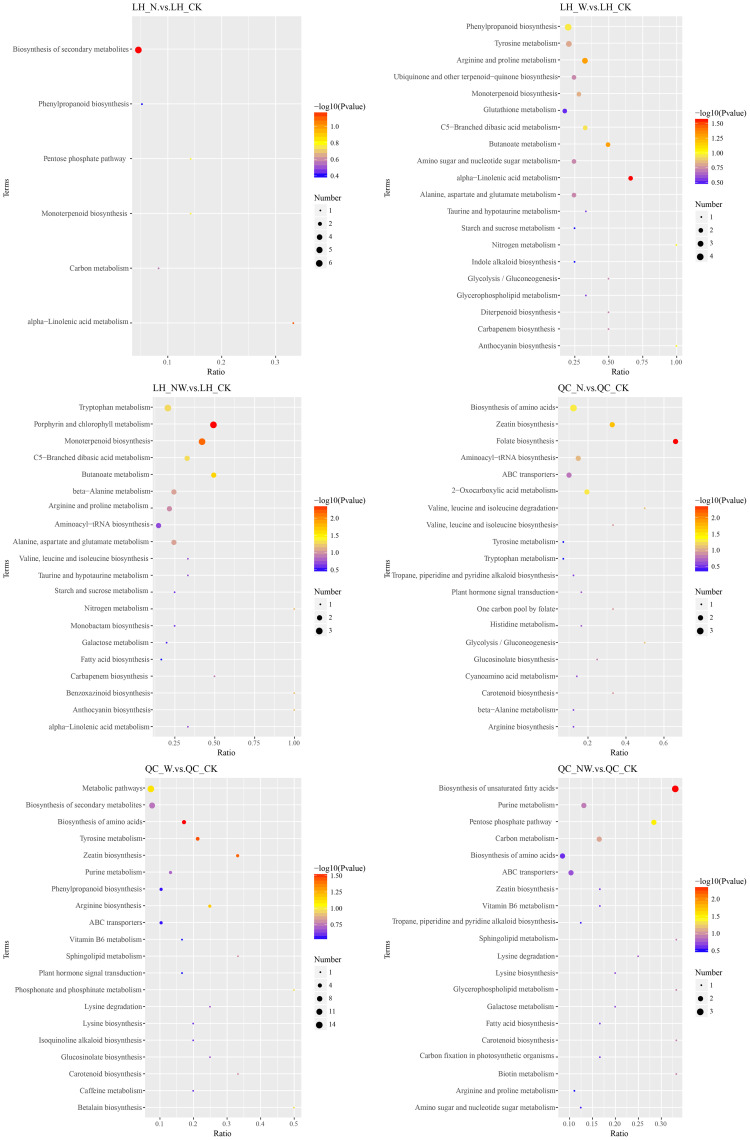
KEGG pathway of differential metabolites in the LH and QC under nitrogen and water addition. LH, *Liquidambar formosana* Hance.; QC, *Quercus acutissima* Carruth.; CK, control; N, canopy N addition at 25 kg ha^−1^ yr^−1^; W, canopy water addition at 30% of the local precipitation; NW, canopy N addition at 25 kg ha^−1^ yr^−1^ and water addition at 30% of the local precipitation. The color of the dot represents the *P*-value. The size of the dot represents the number of differentiated metabolites in the corresponding pathway.

**Figure 9:**
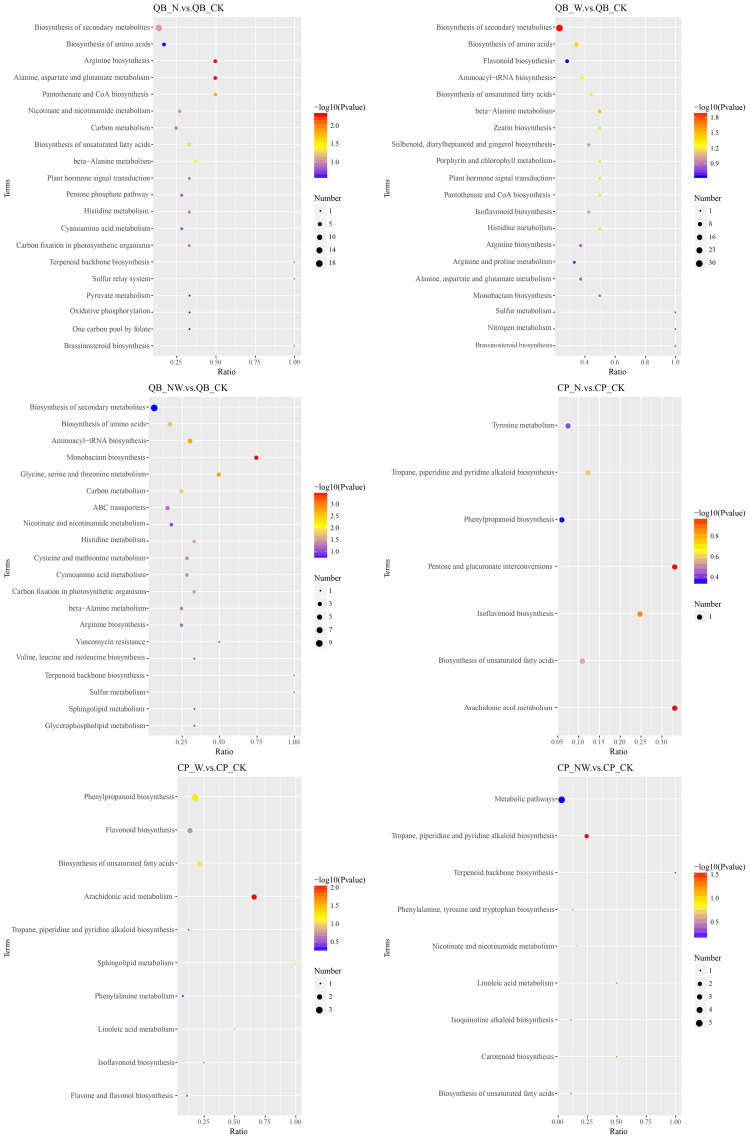
KEGG pathway of differential metabolites in the QB and CP under nitrogen and water addition. QB, *Quercus variabilis* Blume.; CP, *Celtis sinensis* Pers. CK, control; N, canopy N addition at 25 kg ha^−1^ yr^−1^; W, canopy water addition at 30% of the local precipitation; NW, canopy N addition at 25 kg ha^−1^ yr^−1^ and water addition at 30% of the local precipitation. The color of the dot represents the *P*-value. The size of the dot represents the number of differentiated metabolites in the corresponding pathway.

**Figure 10:**
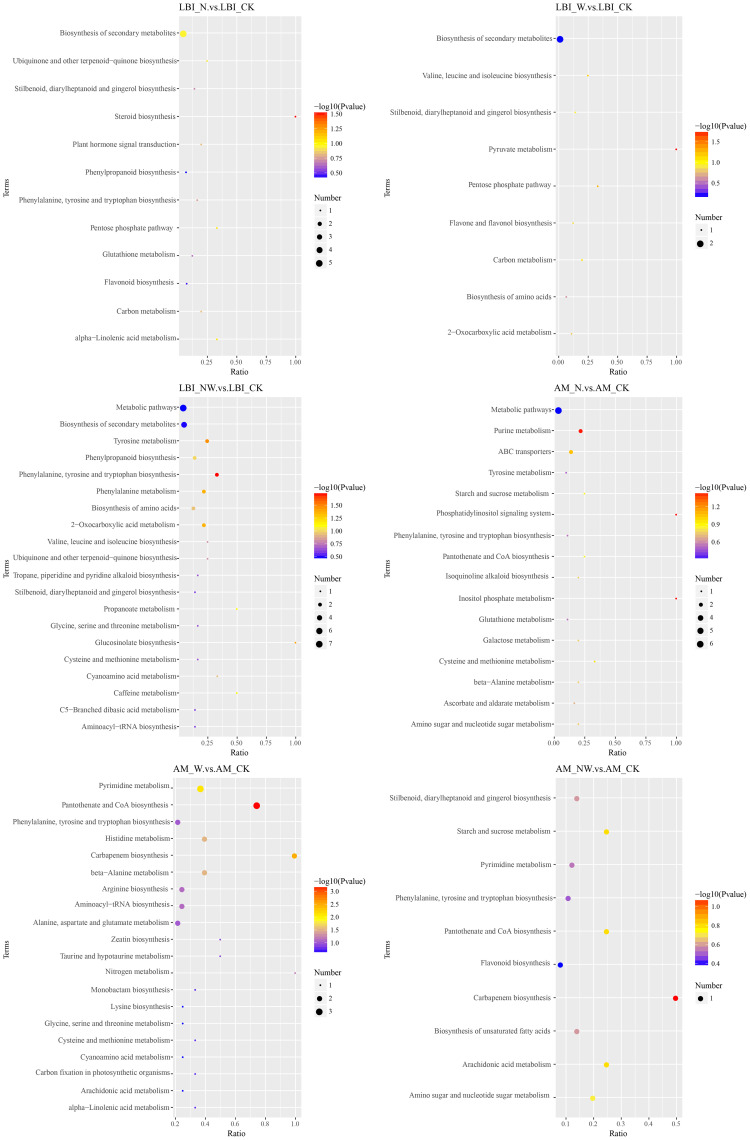
KEGG pathway of differential metabolites in the LBI and AM under nitrogen and water addition. LBI, *Lindera glauca* (Siebold & Zucc.) Blume; AM, *Acer buergerianum* Miq.; CK, control; N, canopy N addition at 25 kg ha^−1^ yr^−1^; W, canopy water addition at 30% of the local precipitation; NW, canopy N addition at 25 kg ha^−1^ yr^−1^ and water addition at 30% of the local precipitation. The color of the dot represents the *P*-value. The size of the dot represents the number of differentiated metabolites in the corresponding pathway.

To further understand the variations in differential metabolites within metabolic pathways, we mapped the identified differential metabolites onto KEGG pathways for annotation and enrichment analyses. Pathways with a significance level of *P*<0.05 were deemed significant metabolic pathways. There were significant changes in 29 metabolic pathways of the three tree species, whereas only 18 metabolic pathways were significantly altered in the three shrub species. In the leaves of the six dominant species, seven pathways were frequently observed across the three comparative pairs: zeatin biosynthesis, terpenoid backbone biosynthesis, pantothenate and CoA biosynthesis, amino acid biosynthesis, beta-alanine metabolism, tyrosine metabolism, and histidine metabolism. These pathways are key metabolic routes through which leaves respond to nitrogen and water treatments (**Figures 8**–**10**).

## 4 Discussion

### 4.1 Variations in morphological and chemical indicators of leaves

In natural ecosystems, nitrogen and precipitation levels are crucial for plant growth and development (Nielsen and Ball, 2015; Zhang et al., 2022) and for leaf traits that reflect plant productivity and carbon balance. SLA, which is influenced by light, moisture, and nutrient conditions, can capture light resources (Ali et al., 2017). The C:N ratio in plants indicates the status of nitrogen supply and fertility levels. In our study involving six species, the SLA of *Quercus variabilis* leaves increased significantly in the N treatment, and the C:N ratio of *Quercus acutissima* leaves notably increased in the NW treatment, with no significant changes observed in the other species. LPC and LMC across the six species remained unchanged in the N and W treatments. These outcomes suggest a certain degree of conservation in foundational leaf traits, which is a result of long-term adaptation to environmental changes. This is consistent with findings from other studies, such as the lack of a significant response in SLA, leaf dry matter content, and leaf carbon content to nitrogen addition in two dominant grassland species (Guo et al., 2022), and the lack of a significant impact on leaf nitrogen concentration following three years of nitrogen and phosphorus addition experiments in tropical forests (Mo et al., 2015).

### 4.2 Responses of leaf physiological processes to nitrogen and water addition

#### 4.2.1 Changes in leaf antioxidant enzymes

Extensive research has shown that the antioxidant capacity of leaves increases significantly when subjected to non-biological stresses (Hossain et al., 2022). Enzymes, such as SOD, POD, and CAT, within plant cells can effectively eliminate reactive oxygen species (Żur et al., 2021), thereby resisting stress. In trees, the POD activity of *Quercus acutissima* leaves was significantly increased in the N and W treatments. In shrubs, the N treatment significantly enhanced the CAT activity of *Acer buergerianum*, the W treatment notably reduced the CAT and POD activities of *Acer buergerianum*, and the NW treatment significantly enhanced the CAT activity of *Lindera glauca*. *Triticum aestivum* can enhance its antioxidant enzyme activity during high-temperature treatments, effectively eliminating reactive oxygen species and preventing lipid peroxidation in cell membranes (Qi et al., 2016). Therefore, changes in enzymatic activity can be used to predict leaf growth.

#### 4.2.2 Changes in leaf osmotic regulating substances

As the level of salt stress increases, the contents of SS, SP, and Pro in the leaves of *Raphanus sativus* seedlings significantly increase (Jia et al., 2020). These compounds are pivotal osmoregulatory substances in plants and play a crucial role in the defense against adverse environmental conditions. Under drought conditions, the Pro content in the leaves of *Pinus massoniana* seedlings increases notably (Wang et al., 2019). The application of nitrogen enhances SP and Pro content in the leaves of dominant grassland species, indicating a high degree of physiological plasticity (Guo et al., 2022). In the case of trees, the N treatment significantly increased the SS content in the leaves of *Liquidambar formosana*, whereas it markedly reduced the SP content. The W treatment significantly increased the Pro content in the leaves of *Quercus variabilis* and notably decreased the SS content in *Quercus acutissima* leaves and the SP content in *Liquidambar formosana* leaves. Among shrubs, the NW treatment significantly elevated the Pro content in *Lindera glauca* and reduced the SS content in *Acer buergerianum*. The findings demonstrate that different species exhibit varied response strategies to nitrogen application and increased precipitation, which actively influence adaptation.

#### 4.2.3 Changes in leaf MDA content

*Avena sativa* seedling root systems exhibit a significant increase in reactive oxygen species when subjected to salt stress. This condition is accompanied by noticeable changes in antioxidant and osmoregulatory substances, along with a substantial increase in MDA content, indicating cellular membrane damage (Gao et al., 2022). Following exposure to high temperatures, *Triticum aestivum* shows a marked increase in the production rate of superoxide anions and hydrogen peroxide content, along with a significant increase in MDA levels (Qi et al., 2016). Interestingly, although the MDA content in *Celtis sinensis* increased significantly under the NW treatment conditions, its antioxidant and osmoregulatory activities remained unchanged. This suggests that *Celtis sinensis* leaves experience stress in the NW treatment, leading to membrane lipid peroxidation. In contrast, other species did not show significant changes in MDA content in the N and W treatments, indicating that their cellular membranes remained intact and that the external stress levels were within their self-regulatory capacity.

### 4.3 Interaction between nitrogen and water addition on forest parameters

In the context of plant biology, C:N, LPC, SLA, and LMC exhibited negligible changes in the N and W treatments. However, significant variations were observed in the levels of antioxidants, osmoregulatory substances, and MDA. These findings indicate that under stress, plants activate osmoregulatory and protective mechanisms, generating compounds that stabilize proteins and prevent oxidative damage (Slama et al., 2015). SEM analysis of trees and shrubs revealed intricate physiological changes in leaf structure. Furthermore, the physical and chemical properties of soil can be influenced by nitrogen addition and water supplementation, affecting plant growth and development (Lu et al., 2014; Wang et al., 2021). Given the simultaneous occurrence of nitrogen deposition and precipitation in the atmosphere, the interaction between the N and W treatments requires further investigation (Cui et al., 2022). The interaction between these treatments significantly affected the Pro content in *Quercus variabilis* leaves, SP and SS content in *Liquidambar formosana* leaves, SS content and POD activity in *Quercus acutissima* leaves, Pro content in *Lindera glauca*, and POD activity in *Acer buergerianum*. The adjustment system of tree leaves underwent more obvious changes than that of shrubs. The FRic index was significantly reduced in the NW treatment, indicating that long-term application of nitrogen and water was not only a nutrient addition but also an environmental stress for the forest, which interfered with the forest production process (Nielsen and Ball, 2015; Zhang et al., 2018). However, there was no significant change in the functional diversity index of the community in other conditions, reflecting the fact that the forest is a complex ecosystem with strong self-regulation ability (Khan et al., 2020; Pierick et al., 2021).

### 4.4 Response of leaf metabolic processes to nitrogen and water addition

#### 4.4.1 Up and downregulated leaf metabolites

When plants are under stress, their metabolic content is upregulated to counteract the stress. Conversely, the metabolic content is downregulated when conditions improve (Fan et al., 2013). Nitrogen addition and increased precipitation significantly changed the contents of various metabolites in the six species. The volcano map showed that the metabolites of the tree leaves were more variable than those of shrub leaves. Differences in metabolic pathways also confirmed that the changes in trees were more significant than those in shrubs. Trees directly receive added nitrogen and water, whereas shrubs tend to absorb nitrogen and water through their roots and soil (Zhu et al., 2019). There were differences in the magnitude of the effects of nitrogen deposition and rainfall on leaf metabolites, and there was an interaction between them. In the W treatment, the frequency of upregulation and downregulation of leaf metabolites was the highest, indicating that the self-regulatory system of leaf metabolites was most active with water addition. Nitrogen deposition and precipitation synergistically alter vegetation growth (Smith et al., 2016). Rainfall enhancement can rapidly increase the amount of water available to plants, improve the osmotic potential of cells, and affect the normal structure and function of plant metabolism. After nitrogen application, plants require a certain amount of time to absorb and assimilate (Lu et al., 2012). These findings are consistent with research indicating that for plant fine roots, the impact of water is more pronounced than that of nitrogen in forests (Li et al., 2023a), corroborating our study findings.

#### 4.4.2 Zeatin biosynthesis pathway

Under the influence of N and W conditions, the zeatin biosynthesis pathway in the leaves of *Quercus acutissima* was significantly enriched, and the dihydrozeatin and adenosine-5′-monophosphate metabolites were upregulated. These results indicate that under conditions of nitrogen application and rainfall enhancement, the leaves can synthesize zeatin to alleviate their stress. Zeatin is a diterpenoid compound that can significantly increase the number of fertile female flowers in *Castanea mollissima*, highlighting its vital regulatory role in female flower formation (Zhou et al., 2023). Furthermore, research on *Oryza sativa* seedlings treated with varying concentrations of a trans-zeatin solution revealed an increase in plant height and stem width, along with an increase in Pro content and decreased glutamate content (Xing et al., 2023). These findings collectively confirm that zeatin is a key metabolite in plant growth.

#### 4.4.3 Terpenoid backbone biosynthesis pathway

Plants produce both primary and specialized terpenoid metabolites (Nagegowda and Gupta, 2020). In the NW treatment, significant changes in the terpenoid backbone biosynthesis pathway were observed in the leaves of *Quercus variabilis* and *Celtis sinensis*, with mevalonic acid being upregulated in *Quercus variabilis* but downregulated in *Celtis sinensis*. Mevalonic acid is involved in a series of enzyme-catalyzed reactions within organisms, contributing to the synthesis of essential precursors of metabolites such as chlorophyll, carotenoids, and diterpene phytoalexins. Terpenoid compounds produced via this pathway can influence multiple aspects of plant growth, development, and stress responses by modulating plant hormone metabolism and phytosterol content (Pu et al., 2021). Phytosterols interact with phospholipids or membrane proteins through their hydroxyl groups to regulate membrane fluidity and permeability. The variation in mevalonic acid content between *Quercus variabilis* and *Celtis sinensis* in the NW treatment highlighted its active role in ensuring normal leaf growth by adapting to environmental changes.

#### 4.4.4 Pantothenate and CoA biosynthesis pathway

In the N treatment, pantothenate and CoA biosynthesis were enriched in *Quercus variabilis*. Metabolites such as L-aspartic acid, pantothenic acid, and uracil were downregulated. Conversely, in the W treatment, this pathway was enriched in *Acer buergerianum*, in which the same metabolites were upregulated. These findings indicate that the pathway actively responds to both N and W treatments, with regulatory strategies varying according to species and treatment conditions. Coenzyme A is a universally present metabolic cofactor that is synthesized from its pantothenic acid precursor (Monteiro et al., 2015; Olzhausen et al., 2021). Coenzyme A plays a crucial role in the synthesis of numerous substances within plants. For instance, under the catalysis of coenzyme A, pantothenic acid is transformed into shikimic acid, a precursor molecule of zeatin, through a series of reactions to ensure the synthesis and stability of zeatin.

#### 4.4.5 Amino acid metabolism pathway

Amino acids play a pivotal role in plant physiology by forming enzymes, hormones, and chlorophyll and by participating in catalytic metabolic processes (The et al., 2020). These processes include growth and development, intracellular pH regulation, generation of redox capacity, and resistance to abiotic and biotic stressors (Hildebrandt et al., 2015; Yang et al., 2019). Amino acids are instrumental in transporting organic nitrogen within plants and serve as cornerstones of nitrogen metabolism. Pathways related to amino acids were frequently annotated in the three comparative pairs in the present study, encompassing the biosynthesis of amino acids, tyrosine metabolism, beta-alanine metabolism, and histidine metabolism. These findings indicate that the synthesis, degradation, and transport of amino acids are modulated in response to environmental changes to meet the internal nitrogen and carbon availabilities of plants, ensuring the maintenance of normal leaf growth (Pratelli and Pilot, 2014). Furthermore, the response strategies of amino acids varied among different treatments and species, consistent with the experimental outcomes observed.

## 5 Conclusion

In this study, the response mechanism of leaves to nitrogen application and rainfall enhancement was analyzed. The results show that levels of antioxidants, osmoregulatory substances, and MDA in the leaves changed significantly, reflecting a stress response that inhibited biomass production. The inhibitory effect on biomass was more pronounced in trees than in shrubs, and water addition imposed greater stress on the leaves than nitrogen addition. Four key metabolic pathways—zeatin biosynthesis, terpenoid backbone biosynthesis, pantothenate and CoA biosynthesis, and amino acid metabolism—were identified as the primary regulatory pathways. With nitrogen and water addition, leaves actively self-regulate to maintain stability in their morphological traits and stoichiometric composition, thereby mitigating the impacts of environmental changes. With increasing nitrogen deposition and shifting precipitation patterns, the regulatory mechanisms in different plant organs must be identified, and the long-term strategies employed by forests to resist environmental stress evaluated. Future studies should move beyond focusing solely on external functional traits and prioritize understanding forest resilience and adaptability across longer timescales under dynamic environmental conditions.

## Data Availability

The original contributions presented in the study are included in the article/**Supplementary Material**. Further inquiries can be directed to the corresponding authors.
