# Supplementary material for: Different responses of canopy and shrub leaves to canopy nitrogen and water addition in warm temperate forest
Source: Front Plant Sci. 2025 Apr 14;16:1530588. doi: 10.3389/fpls.2025.1530588 (PMC12034683; doi:10.3389/fpls.2025.1530588)
Supplement: Supplementary file 1 [file DataSheet1.docx]

**Table S1.** Effects of N, W, N * W on soil physical and chemical properties (*P*-values)

| Treatment | SMC | pH | NO_3_-N | NH_4_-N |
| --- | --- | --- | --- | --- |
| N | 0.992 | 0.499 | 0.738 | 0.818 |
| W | 0.297 | **0.000***** | 0.297 | 0.758 |
| N*W | 0.728 | **0.003**** | 0.196 | 0.428 |

* *P*<0.05, ** *P*<0.01, *** *P*<0.001 in the table indicate significant differences in impact; *P*>0.05 indicates no significant difference. SMC, soil moisture content; NH_4_-N, ammonium nitrogen; NO_3_-N, nitrate nitrogen.

**Table S2.** Statistical table of HMDB metabolites in trees

| Super Class | Number |
| --- | --- |
| Lipids and lipid-like molecules | 264 |
| Phenylpropanoids and polyketides | 184 |
| Organoheterocyclic compounds | 134 |
| Organic acids and derivatives | 125 |
| Benzenoids | 106 |
| Organic oxygen compounds | 93 |
| Nucleosides, nucleotides, and analogues | 35 |
| Lignans, neolignans and related compounds | 15 |
| Alkaloids and derivatives | 11 |
| Organic nitrogen compounds | 8 |
| - | 4 |
| Organosulfur compounds | 1 |

**Table S3.** Statistical table of metabolites of HMDB in shrubs

| Super Class | Number |
| --- | --- |
| Lipids and lipid-like molecules | 381 |
| Phenylpropanoids and polyketides | 246 |
| Organic acids and derivatives | 173 |
| Organoheterocyclic compounds | 164 |
| Benzenoids | 143 |
| Organic oxygen compounds | 133 |
| Nucleosides, nucleotides, and analogues | 43 |
| Alkaloids and derivatives | 33 |
| Lignans, neolignans and related compounds | 18 |
| Organic nitrogen compounds | 12 |
| - | 5 |
| Hydrocarbon derivatives | 1 |
| Organosulfur compounds | 1 |


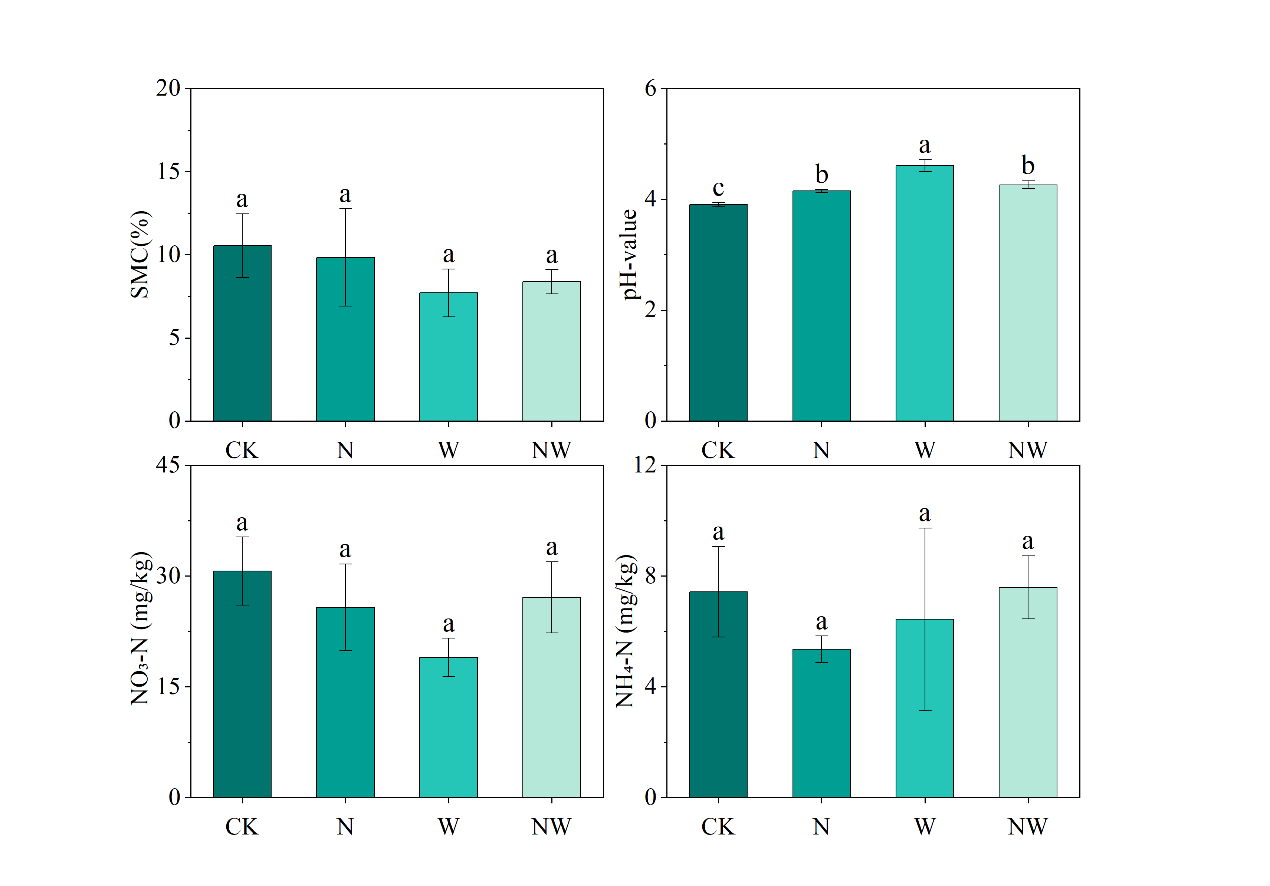


**Figure S1**. Effects of nitrogen and water addition on soil physical and chemical properties. SMC, soil moisture content; NH_4_-N, ammonium nitrogen; NO_3_-N, nitrate nitrogen. CK, control; N, canopy N addition at 25 kg ha^−1^ yr^−1^; W, canopy water addition at 30% of the local precipitation; NW, canopy N addition at 25 kg ha^−1^ yr^−1^ and water addition at 30% of the local precipitation. Different lowercase letters above the error bar (standard error) indicate differences of statistical significance.
